# Bedaquiline pharmacokinetics in drug-resistant tuberculosis: from exposure variability to individualized therapy

**DOI:** 10.3389/fphar.2026.1941682

**Published:** 2026-10-01

**Authors:** Yu Xiong, Jun-Li Wang, Dong-Yan Wei, Yue-Yan Huang, Mao-Shui Wang

**Affiliations:** 1 Department of Tuberculosis, Shandong Public Health Clinical Center, Shandong University, Jinan, China; 2 Guangxi Key Laboratory of Artificial Intelligence for Genetic Diseases of Long-Dwelling Nationalities, Baise, China; 3 Department of Pediatrics, Affiliated Hospital of Youjiang Medical University for Nationalities, Baise, Guangxi, China; 4 Department of Pediatrics, The People’s Hospital of Chongzuo, Chongzuo, Guangxi, China; 5 Department of Lab Medicine, Shandong Public Health Clinical Center, Shandong University, Jinan, China

**Keywords:** bedaquiline, drug–drug interaction, drug-resistant tuberculosis, pharmacokinetics, population pharmacokinetics, precision dosing, therapeutic drug monitoring

## Abstract

Bedaquiline has transformed the treatment of multidrug- and rifampicin-resistant tuberculosis (MDR/RR-TB) and is now a core component of contemporary all-oral regimens. However, translating its distinctive pharmacokinetic properties into individualized clinical management remains challenging. Bedaquiline exhibits food-enhanced absorption, extensive plasma protein binding, broad but heterogeneous tissue distribution, cytochrome P450 3A4-mediated metabolism to the N-monodesmethyl metabolite M2, and prolonged persistence caused by tissue sequestration and slow redistribution. These features contribute to substantial interindividual variability, clinically important drug–drug interactions, delayed accumulation, and residual exposure long after treatment discontinuation. This narrative review synthesizes current evidence on the absorption, distribution, metabolism, and elimination of bedaquiline; population pharmacokinetic models and determinants of exposure variability; target-site penetration; pharmacokinetics in children, pregnancy, organ dysfunction, and HIV coinfection; and clinically relevant interactions with antituberculosis and antiretroviral drugs. We also critically evaluate exposure–efficacy and exposure–toxicity relationships, including evidence from bedaquiline-containing multidrug regimens, and examine the potential roles of therapeutic drug monitoring, minimum inhibitory concentration-normalized exposure indices, and model-informed precision dosing. Current evidence supports administration with food, avoidance of potent *CYP3A* inducers, caution during prolonged *CYP3A* inhibition, and intensified electrocardiographic and electrolyte monitoring when QT-prolonging agents are combined. Nevertheless, no plasma, M2, area-under-the-curve, or susceptibility-normalized exposure target has been prospectively validated for routine dose adjustment. Covariate effects identified by population models are inconsistent across cohorts, and pharmacokinetic evidence remains limited in several special populations. Individualized assessment may therefore be appropriate when clinically meaningful uncertainty exists, but empirical departure from standard dosing is not currently justified. Progress toward precision dosing will require standardized sampling and susceptibility testing, measurement of unbound and target-site exposure, externally validated pharmacokinetic–pharmacodynamic models, and prospective demonstration that exposure-guided interventions improve clinical outcomes.

## 1 Introduction

Multidrug- or rifampicin-resistant tuberculosis (MDR/RR-TB) remains difficult to treat because effective therapy requires prolonged multidrug regimens, sustained adherence and careful management of cumulative toxicity. Historical outcomes illustrate the scale of this challenge. In a systematic review of 74 studies comprising 17,494 patients with microbiologically confirmed pulmonary MDR-TB, treatment success was 60% among patients with MDR-TB and 26% among those with extensively drug-resistant tuberculosis (Bastos et al., 2017). Bedaquiline, a diarylquinoline that selectively inhibits mycobacterial ATP synthase (Andries et al., 2005), has substantially changed the treatment landscape for drug-resistant tuberculosis. Its novel mechanism of action and retained activity against *Mycobacterium tuberculosis* strains resistant to established agents provide an important microbiological basis for its use in combination therapy. Clinical evidence has reinforced this role: In an individual-participant-data meta-analysis of 12,030 patients with MDR-TB from 25 countries, bedaquiline use was associated with a 10-percentage-point higher adjusted probability of treatment success (adjusted risk difference, 0.10; 95% confidence interval [CI], 0.05–0.14) and a 14-percentage-point lower adjusted risk of death (adjusted risk difference, −0.14; 95% CI, −0.19 to −0.10) (Ahmad et al., 2018). Together with its incorporation into World Health Organization (WHO)-recommended all-oral regimens, including bedaquiline–pretomanid–linezolid (BPaL) and bedaquiline–pretomanid–linezolid–moxifloxacin (BPaLM) (WHO consolidated guidelines on tuberculosis, 2025), these findings have established bedaquiline as a backbone component of contemporary RR-TB treatment. Its current prominence therefore reflects the convergence of a distinct mechanism of action, activity against resistant organisms, clinical outcome benefit, and successful integration into shortened multidrug regimens.

The clinical value and complexity of bedaquiline derive from its distinctive pharmacokinetic profile. Oral absorption is slow, and systemic exposure increases with food intake (McLeay et al., 2014). Bedaquiline is highly lipophilic, extensively protein-bound and widely distributed into tissues, and is metabolized predominantly by cytochrome P450 3A4 to the N-monodesmethyl metabolite M2 (van Heeswijk et al., 2014). Although M2 has weaker antimycobacterial activity than the parent compound (Rouan et al., 2012), higher M2 exposure has been associated with corrected QT (QTc) prolongation, whereas its contribution to other toxicities remains uncertain (Tanneau et al., 2021). Bedaquiline and M2 are eliminated mainly in feces, with minimal renal excretion, and exhibit exceptionally long terminal half-lives because of extensive tissue sequestration and slow redistribution (van Heeswijk et al., 2014; Ngwalero et al., 2021). These properties sustain antimycobacterial exposure but also delay accumulation, prolong residual exposure after treatment cessation and increase susceptibility to drug–drug interactions and cumulative toxicity. Exposure varies substantially between individuals and may be influenced by body size, albumin concentration, disease state and concomitant medications. Pharmacokinetic interpretation is therefore central to dose selection, safety monitoring, treatment in special populations and the development of individualized therapeutic strategies.

Previous reviews have addressed selected aspects of bedaquiline pharmacokinetics, drug–drug interactions, pregnancy, and determinants of exposure variability (van Heeswijk et al., 2014; Wilby, 2022; Hughes, 2023). However, a comprehensive, clinically oriented synthesis linking these domains to treatment decisions remains limited. This narrative review synthesizes evidence on bedaquiline absorption, distribution, metabolism and elimination; interindividual variability; special populations; clinically relevant drug–drug interactions; target-site exposure; and pharmacokinetic–pharmacodynamic relationships. It also evaluates the potential roles and current limits of therapeutic drug monitoring and model-informed precision dosing.

## 2 Literature search and evidence selection

We searched PubMed from database inception through 7 July 2026 using combinations of “bedaquiline” with terms related to pharmacokinetics and pharmacodynamics, including absorption, distribution, metabolism, elimination, population pharmacokinetics, exposure, area under the concentration–time curve (AUC), maximum concentration (*C*_max_), minimum concentration (*C*_min_), trough concentration (*C*_trough_), minimum inhibitory concentration (MIC), minimum bactericidal concentration (MBC), tissue or lesion penetration, cerebrospinal fluid, pregnancy, pediatric populations, HIV, drug–drug interactions, therapeutic drug monitoring, and precision dosing. Primary clinical pharmacokinetic and pharmacometric studies in patients with tuberculosis were prioritized, supplemented by relevant translational or preclinical studies when they addressed target-site distribution or mechanisms that could not be evaluated directly in humans. Reference lists of key primary studies and reviews were hand-searched for additional eligible reports. ClinicalTrials.gov was used to verify trial identifiers and current recruitment status, and WHO guidance was used to verify contemporary dosing and programmatic recommendations. Because this is a narrative review, study identification and selection did not follow a formal systematic-review screening or risk-of-bias framework.

## 3 Clinical development and major bedaquiline-containing trials

Bedaquiline development has progressed from add-on evaluation in multidrug-resistant tuberculosis to regimen-level testing in shorter, all-oral combinations. The phase II C208 trial (NCT00449644) and open-label C209 study (NCT00910871) generated foundational efficacy, safety, and pharmacometric data. DELIBERATE (NCT02583048) evaluated bedaquiline, delamanid, and their combination, including in participants with HIV. Nix-TB (NCT02333799) and ZeNix (NCT03086486) evaluated BPaL, whereas TB-PRACTECAL (NCT02589782) and endTB (NCT02754765) tested shorter bedaquiline-containing all-oral regimens across broader drug-resistant tuberculosis populations.

More recent studies extend these questions to specific populations and treatment strategies. BEAT-TB India (CTRI/2019/01/017310) generated intensive pharmacokinetic data in adults with pulmonary pre-extensively drug-resistant tuberculosis receiving bedaquiline, delamanid, linezolid, and clofazimine. As of 29 August 2026, C211 (NCT02354014), PRISM-TB (NCT06441006), and QUANTUM-TB (NCT07209761) were listed as recruiting. These studies address pediatric pharmacokinetics, stratified shortened BPaLM strategies, and quabodepistat-containing regimens, respectively. Selected pivotal and active trials are summarized in Supplementary Table S1.

## 4 Core pharmacokinetic characteristics of bedaquiline

The integrated pharmacokinetic disposition of bedaquiline and its clinical implications are summarized in Figure 1.

**FIGURE 1:**
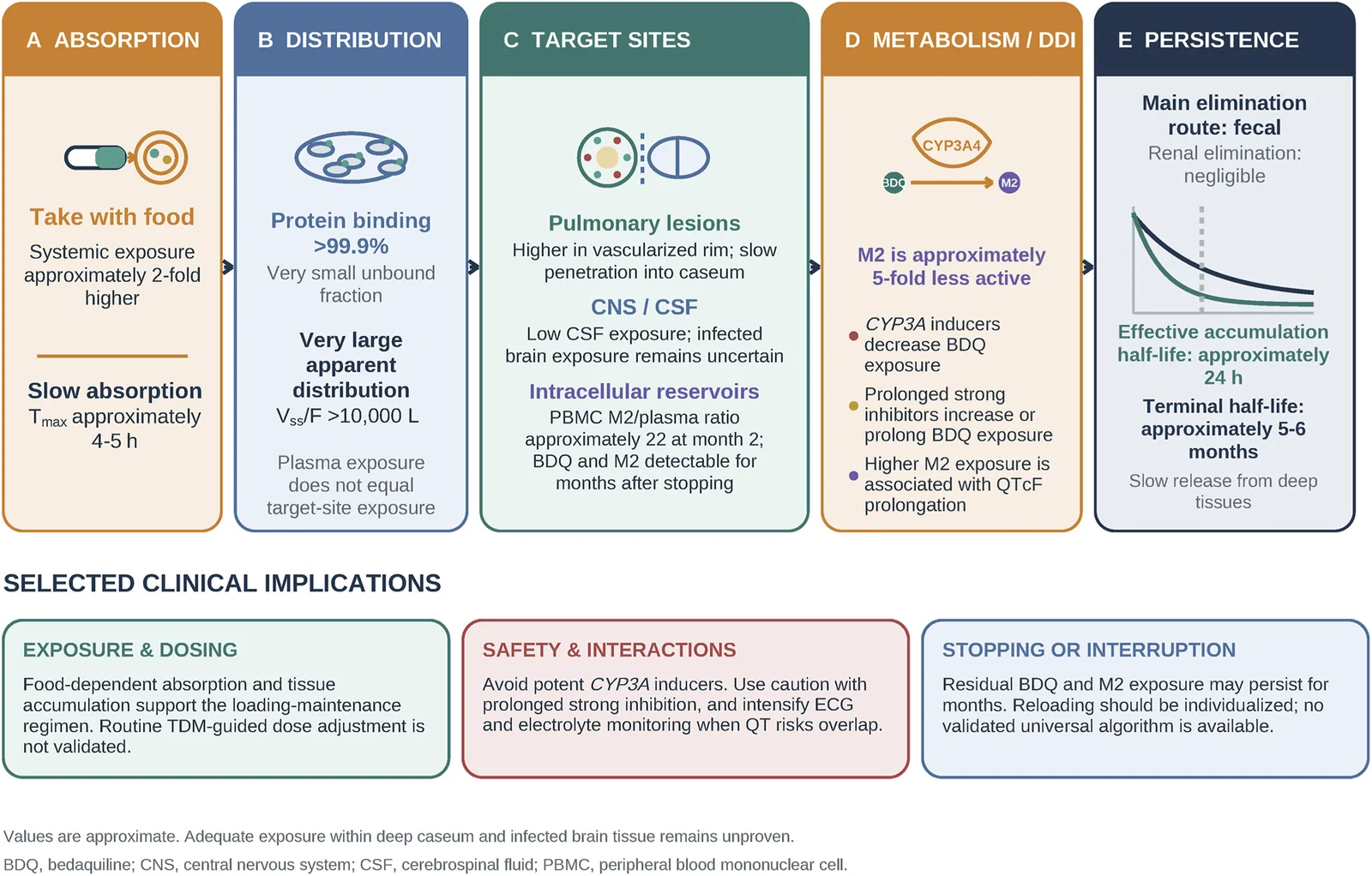
Integrated pharmacokinetic disposition of bedaquiline and its clinical implications.

### 4.1 Absorption and food effect

Bedaquiline is absorbed slowly after oral administration, with maximum plasma concentrations typically reached 4–5 h after dosing (McLeay et al., 2014; Rustomjee et al., 2008). In an early dose-ranging study, *C*_max_ and AUC increased approximately proportionally across doses of 25–400 mg, whereas the time to maximum plasma concentration (*T*_max_) remained close to 4 h, indicating that dose primarily altered the extent rather than the rate of exposure (Rustomjee et al., 2008). Population pharmacokinetic analyses have subsequently used delayed or multiple-input models to describe the complex absorption profile (McLeay et al., 2014).

Food markedly increases systemic exposure. In healthy participants, administration with food increased bedaquiline AUC by approximately 2.0- to 2.4-fold compared with fasting administration (van Heeswijk et al., 2014). A later bioequivalence study in healthy Chinese adults similarly showed substantially higher exposure under postprandial conditions, although it was not designed as a within-participant food-effect comparison (Li et al., 2025). This reproducible effect makes administration with food an integral component of dosing rather than merely a measure to improve gastrointestinal tolerability.

### 4.2 Distribution, extensive protein binding and tissue sequestration

Bedaquiline is highly lipophilic and more than 99.9% bound to plasma proteins (van Heeswijk et al., 2014; Ngwalero et al., 2021). Population pharmacokinetic models estimate an apparent steady-state volume of distribution exceeding 10,000 L, indicating extensive partitioning beyond the vascular compartment (McLeay et al., 2014). This exceptionally large apparent volume reflects sequestration within peripheral tissues and intracellular compartments rather than unrestricted distribution of pharmacologically active unbound drug.

Clinical measurements support substantial intracellular accumulation. In patients with rifampicin-resistant tuberculosis, plasma and peripheral blood mononuclear cell exposures to parent bedaquiline were broadly comparable, whereas intracellular M2 exposure markedly exceeded plasma exposure. The estimated intracellular-to-plasma concentration ratio for M2 increased during the first 2 months of treatment and reached approximately 22 (Ngwalero et al., 2021). However, peripheral blood mononuclear cell concentrations should not be assumed to represent exposure within pulmonary lesions or other target tissues.

Distribution within pulmonary lesions is spatially heterogeneous. In cavitary tuberculosis models, bedaquiline concentrations were highest in the vascularized cellular rim but declined with increasing distance into the avascular caseous core (Bustion et al., 2025). Penetration into deep caseum was slow, and concentrations in poorly vascularized regions required weeks to months to approach a plateau. High plasma or cellular exposure therefore does not necessarily indicate rapid or uniform penetration throughout the lesion. This spatial and temporal mismatch may also have implications for resistance selection. Translational lesion studies suggest that slowly rising concentrations in deep caseum, followed by prolonged declining concentrations after treatment, can create periods in which bacilli are exposed to drug concentrations below those associated with maximal activity (Bustion et al., 2025). This represents a plausible window for selection of resistant subpopulations, although the magnitude of this risk has not been prospectively quantified in patients.

Cerebrospinal fluid exposure is also limited. An initial case report of multidrug-resistant tuberculous meningitis found no quantifiable bedaquiline in cerebrospinal fluid (Akkerman et al., 2016). In a subsequent study of seven patients, mean cerebrospinal fluid-to-plasma concentration ratios for bedaquiline and M2 were only 0.12% and 0.30%, respectively (Upton et al., 2022). These ratios approximated the estimated unbound plasma fractions, suggesting that cerebrospinal fluid exposure largely reflects the very small circulating unbound fraction. In BALB/c mice, the brain-to-plasma AUC ratio for bedaquiline was 0.20, compared with 11.95 in lung tissue (Zhu et al., 2026). These limited data should not be interpreted as definitive measures of exposure in infected human brain tissue. Overall, available evidence indicates low and variable cerebrospinal fluid exposure and does not establish adequate penetration into infected brain tissue. Data remain insufficient for bone, pleural tissue and other anatomically protected sites.

### 4.3 *CYP3A4*-mediated metabolism and preclinical evidence on M2

Bedaquiline undergoes predominantly cytochrome P450 3A4-mediated N-demethylation to form its principal circulating metabolite, N-monodesmethyl bedaquiline (M2) (van Heeswijk et al., 2014). This pathway underlies the susceptibility of bedaquiline exposure to *CYP3A4* induction and inhibition, considered in Section 6.

Preclinical studies indicate that M2 retains antimycobacterial activity but is substantially less potent than the parent compound. *In vitro*, bedaquiline was approximately fivefold more active against *Mycobacterium tuberculosis* than M2, and substantial M2-mediated bacterial killing in murine models required exposures well above those generally achieved clinically (Rouan et al., 2012). M2 is therefore unlikely to contribute substantially to the therapeutic activity of standard bedaquiline-containing regimens.

Preclinical toxicology has also identified potential M2-related toxicity at high exposure. In long-term dog studies, dose- and time-dependent phospholipidosis occurred predominantly in cells of the mononuclear phagocyte system, with M2 identified as the principal contributor to toxicity at high exposures (Smyej et al., 2017). Neither finding was observed at exposures approximating therapeutic human exposure. These findings do not imply that clinically relevant exposure inevitably causes phospholipidosis, but they identify M2 as a potential determinant of tissue toxicity under excessive exposure. Human exposure–toxicity evidence, particularly the relationship between M2 and QT prolongation, is discussed separately in Section 9.2 (Tanneau et al., 2021).

### 4.4 Elimination, prolonged half-life and accumulation

Bedaquiline and its metabolites are eliminated predominantly in feces, whereas renal excretion of unchanged bedaquiline is negligible (van Heeswijk et al., 2014). The defining feature of elimination is the slow return of drug from deeply distributed tissue compartments to the circulation. This distribution-limited process produces a multiphasic concentration–time profile and an exceptionally prolonged terminal phase (McLeay et al., 2014; van Heeswijk et al., 2014).

The terminal half-life of bedaquiline is approximately 5–6 months (van Heeswijk et al., 2014; Keutzer et al., 2022). This estimate primarily reflects tissue sequestration and slow redistribution rather than unusually slow hepatic clearance. Consistent with this mechanism, bedaquiline remained quantifiable in plasma 200 days after treatment discontinuation in one patient (Perrineau et al., 2019), and both bedaquiline and M2 remained detectable in plasma and peripheral blood mononuclear cells for up to 6 months after cessation in another cohort (Ngwalero et al., 2021).

The terminal half-life should be distinguished from the effective half-life governing accumulation during repeated dosing. Multiple-dose data suggest an effective accumulation half-life of approximately 24 h despite a terminal half-life of about 5.5 months (Keutzer et al., 2022). The effective half-life reflects the rate at which plasma exposure approaches its treatment-phase plateau, whereas the terminal half-life describes persistence during the prolonged post-distribution phase. Conflating these measures would substantially overestimate the time required for clinically relevant exposure to develop. Tissue sequestration therefore sustains exposure after dosing but also produces gradual accumulation and prolonged residual concentrations, complicating treatment interruption.

## 5 Dosing regimens and exposure measures

### 5.1 Dosing schedules, accumulation, and treatment interruption

Current WHO guidance recognizes two bedaquiline schedules used in BPaLM/BPaL for adults and adolescents aged ≥14 years (WHO consolidated guidelines on tuberculosis, 2025). The conventional schedule is 400 mg once daily for 2 weeks followed by 200 mg three times weekly; the alternative schedule, evaluated in ZeNix, is 200 mg once daily for 8 weeks followed by 100 mg once daily. The schedules differ in dosing frequency and therefore in the interpretation of short-interval concentrations and AUC measures.

With once-daily dosing, the dosing interval is 24 h, so AUC_0–24h_ represents full-interval exposure and an immediately pre-dose sample can represent *C*_trough_. During three-times-weekly maintenance dosing, intervals are typically 48 or 72 h; AUC_0–24h_ then represents only part of the interdose exposure, and a concentration measured 24 h after a dose is not a true trough. Exposure metrics should therefore be interpreted in relation to the actual dosing and sampling interval.

Bedaquiline concentrations continue to evolve as peripheral tissue compartments equilibrate (McLeay et al., 2014). Model-based analyses suggest that reloading after an interruption should depend on its timing and duration; repeating the full 2-week loading phase after a shorter interruption may produce excessive M2 exposure (Keutzer et al., 2022). These simulations have not yielded a prospectively validated universal reloading algorithm.

### 5.2 Exposure measures and cross-schedule

Bedaquiline exposure is described using *C*_max_, *C*_min_, *C*_trough_, and AUC, but these measures are not interchangeable. *C*_max_ is the maximum concentration. *C*_min_ is the minimum observed or model-estimated concentration within a specified interval and should not automatically be called a trough. *C*_trough_ is the concentration immediately before the next scheduled dose. AUC_0–24h_ quantifies exposure during the first 24 h after dosing, whereas AUC_0–τ_ covers the complete dosing interval (τ).

Under once-daily dosing, AUC_0–24h_ is equivalent to AUC_0–τ_. Under three-times-weekly maintenance dosing, full-interval exposure should instead be expressed over the relevant 48- or 72-h interval; standardized cumulative exposure such as AUC_0–168h_ can be useful for cross-schedule comparisons. Reported metrics should retain the definitions used in the source study rather than be retrospectively relabeled. This distinction is illustrated by a small phase 2 study in Japanese adults with pulmonary MDR-TB: bedaquiline AUC_0–24h_ ranged from 50.6 to 107.3 mg·h/L at week 2 during once-daily loading (n = 5), whereas values of 58.5 and 77.1 mg·h/L were observed in two participants at week 24 during three-times-weekly maintenance. The latter values therefore represent 24-h post-dose exposure rather than complete interdose exposure and should not be interpreted as directly comparable full-interval AUCs (Tsuyuguchi et al., 2019).

In a separate prospective cohort, median bedaquiline *C*_max_, *C*_min_, and AUC_0–24h_ were 2.03 mg/L, 0.68 mg/L, and 30.6 mg·h/L, respectively (Alghamdi et al., 2021). These are descriptive study-specific estimates, not universal reference concentrations or validated therapeutic targets.

Susceptibility-normalized indices such as AUC/MIC and *C*_max_/MIC relate systemic exposure to the minimum inhibitory concentration of the infecting isolate. Their associations with microbiological outcomes are discussed in Section 9 and their potential role in dose optimization in Section 10.

In a small retrospective cohort receiving prolonged bedaquiline treatment, the pooled median M2-to-bedaquiline concentration ratio was 0.15 (interquartile range [IQR], 0.10–0.25) (Perrineau et al., 2019). Because sampling times were heterogeneous, this ratio should be treated as descriptive rather than as a reference range or validated efficacy or toxicity marker. Additional context-specific pharmacokinetic estimates are provided in Supplementary Table S2.

## 6 Exposure variability and population pharmacokinetic studies

### 6.1 Overview of population pharmacokinetic models

Population pharmacokinetic modeling has been central to characterizing bedaquiline because its multiphasic disposition and prolonged terminal phase are poorly captured by conventional noncompartmental analyses. The first comprehensive model by McLeay et al. incorporated 5,222 plasma concentrations from 480 participants enrolled across the phase I/II bedaquiline development program, including healthy volunteers, patients with drug-susceptible tuberculosis, and participants from the C208 and C209 MDR-TB studies (McLeay et al., 2014). Importantly, subsequent models were not based on entirely independent populations. Svensson et al. reanalyzed data from C208 (NCT00449644) and C209 (NCT00910871), including 335 patients with drug-resistant tuberculosis, to jointly characterize bedaquiline and M2 and evaluate longitudinal changes in body weight and albumin (Svensson et al., 2016a). Later population models were predominantly developed from separate clinical cohorts in China and other settings, generally with smaller and less intensively sampled populations (Zhu et al., 2021; Zou et al., 2022; Shao et al., 2023). The source populations and extent of dataset overlap are summarized in Table 1.

**TABLE 1:** Key clinical and translational evidence on bedaquiline pharmacokinetics and exposure variability^a,b^.

Study and population	Source dataset/parent study	Design	Key findings	Clinical interpretation	Main limitations
A. Clinical and population pharmacokinetic studies					
(McLeay et al., 2014)^c^ Healthy volunteers and people with tuberculosis; n = 480; 5,222 observations	Pooled phase I/II BDQ development program, including C208 stages 1/2 and C209, together with phase I healthy-volunteer and phase IIa DS-TB studies	Parent-drug PopPK; 4-compartment disposition with dual zero-order inputs	CL/F was 2.78 L/h and apparent V_ss_/F exceeded 10,000 L. Study-reported Black race was associated with 52% higher CL/F; female sex with 15.7% lower central V/F	Established the multiphasic disposition model. The demographic effects were not considered sufficient for routine dose adjustment	M2 was not modeled; heterogeneous pooled phase I/II data; limited long-term sampling; partly overlaps with later C208/C209 analyses
(Svensson et al., 2016a)^c^ People with MDR-TB receiving 24 weeks of BDQ; n = 335	C208 (NCT00449644) and C209 (NCT00910871): 23 C208 stage 1, 79 C208 stage 2, and 233 C209 participants	Joint BDQ-M2 PopPK with longitudinal weight and albumin covariates	The included covariates jointly reduced between-person variability in clearance from 62% to 48% for BDQ and from 72% to 60% for M2. Age and study-reported Black race were also associated with clearance	Recovery-related changes in weight and albumin affect exposure estimates, but no covariate supports a validated dosing rule	Complex model; external validation limited; requires longitudinal covariate data; Not independent of the C208/C209 data contributing to McLeay et al.
(Zhu et al., 2021) Chinese adults with DR-TB; n = 69; 306 concentrations	Independent Chinese clinical cohort; not derived from C208/C209	Parent-drug PopPK; 3-compartment model with transit absorption	Typical CL/F was 1.50 L/h with 65% interindividual variability. Baseline age, sex, body weight, hepatic and renal measures, and electrolytes did not significantly affect CL/F	Illustrates substantial unexplained variability and the inconsistency of proposed clinical covariates across cohorts	Single-country sparse dataset; parent drug only; limited external validation
(Alghamdi et al., 2021) Prospective Georgian DR-TB cohort; BDQ analysis n = 63	Prospective routine-treatment cohort at the National Center for Tuberculosis and Lung Disease, Tbilisi, Georgia	Semi-intensive 24-h sampling at 4–6 weeks; noncompartmental and covariate analyses	Median *C*_min_ was 0.68 mg/L and AUC_0-24h_ was 30.6 mg⋅h/L. Associations with sex, weight, and creatinine clearance were exploratory	Confirms marked real-world exposure variability; the reported concentrations are descriptive, not therapeutic targets	Small subgroup; limited absorption-phase and terminal sampling; observational covariate analysis
(Ngwalero et al., 2021) South African adults with RR-TB; n = 21	Prospective South African PK cohort; serial plasma/PBMC sampling during and after treatment	Plasma and PBMC PK during treatment and for 6 months after stopping; effect-compartment model	At month 2, PBMC-to-plasma ratios were 1.10 for BDQ and 22.2 for M2. Both compounds remained detectable in PBMCs 6 months after treatment	Supports a slowly exchanging intracellular reservoir, especially for M2; plasma M2 may underrepresent PBMC exposure	Small cohort; only one plasma sample and two PBMC samples were available 6 months after treatment completion; total concentrations only; PBMCs are not surrogates for pulmonary lesions
Pharmacogenetic studies (Zou et al., 2022; Haas et al., 2022) Chinese and South African DR-TB cohorts	Independent Chinese and South African clinical PK/pharmacogenetic cohorts	Candidate pharmacogenetic covariates within PopPK analyses	*AGBL4* rs319952 GG and higher GGT were associated with lower clearance in China; CYP3A5*3 was associated with approximately 15%–30% slower clearance in South Africa	Genetic and biochemical factors may explain a limited fraction of variability, but routine genotype-guided dosing is not justified	Cohort-specific findings; modest power; parent-drug models; independent replication and prospective validation lacking
B. Target-site distribution and translational models					
(Bustion et al., 2025) Rabbit cavitary-TB site-of-disease PK linked to human simulations	Rabbit cavitary-TB site-of-disease data linked to clinical plasma PK models	Laser-capture microdissection and spatial caseum diffusion modeling	BDQ accumulated near the vascularized rim but reached efficacious concentrations in deep caseum only after weeks to months; subtherapeutic concentrations could persist long after treatment	High plasma or cellular exposure does not ensure rapid deep-caseum exposure; slow diffusion may create windows of suboptimal lesion concentrations	Rabbit lesion data extrapolated to humans; results depend on caseum PK-PD targets and lesion assumptions
(Upton et al., 2022) Adults with RR pulmonary TB without CNS disease; n = 7	Prospective clinical PK study in adults with RR pulmonary TB	Steady-state plasma PK with one matched lumbar CSF sample per participant	Mean CSF-to-plasma concentration ratios were 0.12% for BDQ and 0.30% for M2; CSF concentrations approximated the estimated unbound plasma fractions	Provides direct human evidence of very low total CSF exposure, but does not establish exposure in infected brain tissue or efficacy in TB meningitis	All participants were male and had no CNS TB; one CSF sample was obtained per participant; unbound concentrations were estimated rather than directly measured
(Mehta et al., 2023) Translational mouse-to-human lung-lesion simulations	Translational preclinical-to-human lung/lesion mPBPK dataset/model	Minimal PBPK model with virtual-patient PTA analysis	More than 80% were predicted to attain whole-lung targets, whereas fewer than 5% attained the nonreplicating-bacilli target in caseous lesions during maintenance dosing	Whole-lung exposure may substantially overstate exposure in avascular caseum	No direct human BDQ lesion concentrations; cross-species translation and *in vitro* targets
(Mehta et al., 2024a) Human plasma and sparse CSF data	Human plasma PK and sparse CSF data used to evaluate a whole-body CNS PBPK model	Whole-body PBPK model with CSF, brain interstitial, and intracellular compartments	Predicted unbound brain interstitial concentrations approximated unbound plasma concentrations, whereas predicted unbound brain intracellular-to-plasma ratios were approximately 0.07 for BDQ and 0.03 for M2. Intracellular brain exposure remained substantially lower than intracellular lung exposure	Low total CSF concentrations do not necessarily indicate absent unbound interstitial exposure; however, the model predicts limited intracellular exposure at the presumed CNS site of infection, and clinical efficacy in tuberculous meningitis remains unvalidated	Sparse CSF data; no infected-brain tissue validation; binding assumptions drive predictions
(Karakitsios et al., 2025) Cross-species preclinical data extrapolated to human lung lesions	Preclinical mouse, rat, and dog plasma/lung PK datasets plus published mouse lesion data; human PopPK model used for extrapolation	PBPK model with macrophage lysosomal trapping and spatial caseum diffusion	Predicted high macrophage accumulation but limited penetration into the central caseous core	Explains why extensive cellular accumulation can coexist with poor deep-lesion penetration	Sparse human lesion data; structural and binding assumptions; not a clinical dosing algorithm
C. Special populations and organ impairment					
(Hughes et al., 2022) Children and adolescents aged 6–17 years with MDR/RR-TB; n = 15	Independent observational pediatric MDR/RR-TB PK cohort	Semi-intensive PK and safety study; adult PopPK model with allometric scaling	Estimated bioavailability was 57% of the adult value; only 3 of 15 attained the prespecified adult weekly AUC reference. Two mild QTcF prolongations occurred	Supports pediatric exposure bridging rather than simple proportional dose reduction; does not establish exposure-guided escalation	Very small, predominantly adolescent cohort; no children younger than 6 years; limited covariate power
(Court et al., 2022) Pregnant women with RR-TB and HIV; n = 13; paired profiles n = 6	Longitudinal PK substudy nested within a South African cohort of pregnant women with RR-TB	Third-trimester and postpartum PK with breast-milk and infant sampling	Maternal BDQ and M2 concentrations were about 50% below nonpregnant predictions in participants not receiving LPV/r. Milk-to-plasma accumulation ratios were 13.6 for BDQ and 4.84 for M2	Suggests possible maternal underexposure and substantial infant exposure, but does not support a pregnancy-specific dose recommendation	Small cohort; all participants had HIV; sparse sampling; pregnancy and postpartum effects not reliably separated

^a^ Clinical rows summarize observed data; target-site rows include model-based predictions.

^b^ Model-derived covariates and candidate thresholds are hypothesis-generating and should not be interpreted as validated dose-adjustment rules.

^c^ Some pharmacometric analyses used overlapping source populations. In particular, C208 and C209 contributed to both the McLeay et al. (2014) and Svensson et al. (2016a) models; these analyses should therefore not be interpreted as independent replication.

Abbreviations: AUC, area under the concentration-time curve; BDQ, bedaquiline; CL/F, apparent oral clearance; *C*_max_, maximum concentration; *C*_min_, minimum concentration; CNS, central nervous system; CSF, cerebrospinal fluid; DR-TB, drug-resistant tuberculosis; GGT, gamma-glutamyl transferase; HIV, human immunodeficiency virus; LPV/r, lopinavir/ritonavir; M2, N-monodesmethyl bedaquiline; mPBPK, minimal physiologically based pharmacokinetic; MIC, minimum inhibitory concentration; MDR-TB, multidrug-resistant tuberculosis; PBMC, peripheral blood mononuclear cell; PBPK, physiologically based pharmacokinetic; PK, pharmacokinetic; PK–PD, pharmacokinetic–pharmacodynamic; PopPK, population pharmacokinetic; PTA, probability of target attainment; QTcF, Fridericia-corrected QT, interval; RR-TB, rifampicin-resistant tuberculosis; V/F, apparent volume of distribution; Vss/F, apparent steady-state volume of distribution.

Despite differences in model structure and source population, several features were common across the major bedaquiline population pharmacokinetic analyses. Most were developed primarily from plasma concentration data in adults with drug-resistant tuberculosis and used compartmental population models to characterize substantial between-patient variability in exposure. Clinical or physiological covariates were routinely explored as potential determinants of clearance or distribution. However, the models differed markedly in structural complexity, with one to four disposition compartments and first-order, transit, or dual-input absorption models. M2 was incorporated inconsistently, and most studies quantified total rather than unbound drug concentrations. Independent external validation was also uncommon. Thus, the common feature across models is not a single structural model or reproducible covariate set, but rather the consistent finding that bedaquiline exhibits complex multiphasic disposition and considerable interindividual variability. These similarities support population modeling as an appropriate framework for characterizing bedaquiline pharmacokinetics, whereas the structural and population differences caution against directly transferring parameter estimates or dosing implications from one model to another. The principal pediatric study adapted an adult model through allometric scaling in only 15 children and adolescents (Hughes et al., 2022), and models in patients with specific comorbidities have generally been derived from small, geographically restricted cohorts.

### 6.2 Covariate evaluation and cross-model consistency

Covariate effects on bedaquiline pharmacokinetics have varied substantially across studies, with body size and albumin receiving the most consistent attention. In the joint bedaquiline–M2 population pharmacokinetic model, typical body weight increased from 56.6 kg at baseline to 62.6 kg at week 120, while albumin increased from 3.65 g/dL toward 4.04 g/dL (Svensson et al., 2016a). Time-varying weight influenced clearance and distribution volumes, whereas albumin affected the disposition of both bedaquiline and M2. Incorporating these longitudinal changes reduced unexplained between-patient variability in bedaquiline clearance from 62% to 48%. A separate model incorporated allometric scaling and identified albumin as a significant covariate of clearance (Shao et al., 2023). Consistent with a potential body-size effect, a prospective cohort found that each 10-kg increase in body weight was associated with an approximately 0.12-mg/L decrease in bedaquiline *C*_min_ (Alghamdi et al., 2021). However, these findings have not been reproduced uniformly. In another Chinese population pharmacokinetic model, baseline body weight and the evaluated biochemical variables were not significantly associated with clearance (Zhu et al., 2021). In a small retrospective analysis, bedaquiline concentrations were positively correlated with C-reactive protein, although no bedaquiline-specific multivariable predictor was identified (Quncuo et al., 2025).

Demographic effects have been similarly inconsistent. In the initial population pharmacokinetic analysis, Black participants had 52% higher apparent clearance, whereas women had a 15.7% lower central distribution volume (McLeay et al., 2014); neither effect was considered sufficient to justify routine dose modification. In the joint bedaquiline–M2 model, Black race was again associated with higher clearance and increasing age with lower clearance, whereas sex, HIV infection, and extensively drug-resistant tuberculosis were not significant covariates (Svensson et al., 2016a). By contrast, a Chinese model found no significant effects of age, sex, body weight, hepatic function, or renal function on apparent clearance (Zhu et al., 2021). In a prospective clinical cohort, male sex was associated with higher *C*_min_ and AUC, increasing age with higher *C*_min_, and greater creatinine clearance with lower AUC (Alghamdi et al., 2021). These associations were exploratory and derived from a relatively small cohort, limiting their use for clinical dose individualization.

Genetic, biochemical, and disease-related covariates have also been explored, but evidence remains cohort-specific. In South African patients, CYP3A5*3 heterozygosity and homozygosity were associated with approximately 15% and 30% slower bedaquiline clearance, respectively (Haas et al., 2022). A Chinese population pharmacokinetic model identified gamma-glutamyl transferase and an *AGBL4* polymorphism as clearance covariates (Zou et al., 2022). Neither finding has established a reproducible pharmacogenetic or biochemical dosing strategy. Similarly, a population model in 58 patients with MDR-TB and type 2 diabetes could not determine whether diabetes independently altered bedaquiline exposure because no nondiabetic comparator group was included (Cao et al., 2025).

Taken together, no covariate has shown sufficiently consistent effects across populations to support routine covariate-based bedaquiline dose adjustment. Body weight and albumin have the strongest repeated physiological support, but their effects are not uniform across models. Associations with age, sex, race, renal function, inflammatory or biochemical markers, and genetic variants are less consistent or remain cohort-specific. Moreover, because bedaquiline is more than 99.9% protein-bound, changes in total plasma concentrations associated with albumin or other physiological factors may not translate proportionally into changes in unbound or target-site exposure. Current covariates may therefore improve population-level model fit or help explain exposure variability, but none should presently be used as a stand-alone clinical dosing rule.

### 6.3 Physiologically based and translational pharmacokinetic models

Physiologically based and translational models have extended plasma pharmacokinetics to target-site exposure. One model predicted that more than 80% of patients receiving standard dosing would attain lung concentrations above the bactericidal target for replicating bacilli, whereas fewer than 5% would attain caseous-lesion concentrations above the target for nonreplicating bacilli during maintenance therapy (Mehta et al., 2023).

A whole-body model predicted unbound brain interstitial concentrations comparable to unbound plasma concentrations, but unbound intracellular brain concentrations only approximately 7% of those in lung cells (Mehta et al., 2024a). Cross-species lung modeling attributed extensive cellular accumulation to lysosomal ion trapping and predicted limited penetration into the central caseous core (Karakitsios et al., 2025). These models explain why plasma exposure may diverge from target-site exposure, but their predictions depend on assumptions about tissue binding, lesion structure, diffusion and pharmacodynamic targets and should not guide dose adjustment without validation.

### 6.4 Limitations of current population pharmacokinetic evidence

The evidence base remains constrained by population selection, sampling and model heterogeneity. The largest datasets arose from controlled adult trials, whereas many later studies included fewer than 100 participants from single countries or centers. Children, pregnant and postpartum women, patients with major organ dysfunction and those with complex comorbidities remain underrepresented.

Models ranging from one to four compartments and using first-order, transit-compartment or dual-input absorption have adequately described their respective datasets (McLeay et al., 2014; Svensson et al., 2016a; Zhu et al., 2021; Zou et al., 2022; Shao et al., 2023), limiting direct comparison of parameters. Sparse sampling and the prolonged terminal phase impair estimation of peripheral distribution. M2 has not been modeled consistently, and most studies measured total rather than unbound concentrations despite extensive protein binding. External validation is limited, and covariate effects frequently differ between populations. Routine therapeutic drug monitoring data may broaden real-world evidence but are vulnerable to heterogeneous sampling, adherence and referral bias.

The availability of multiple population pharmacokinetic models should not be interpreted as evidence that they are interchangeable for clinical decision-making. Model selection should depend on the intended clinical question and on the similarity between the individual patient and the population in which the model was developed. Relevant considerations include age and disease setting, dosing schedule and treatment phase, source and density of pharmacokinetic sampling, whether both bedaquiline and M2 were modeled, the covariates incorporated, and whether the model has undergone independent external validation. Models developed in large trial datasets may provide more stable structural estimates, whereas smaller cohort-specific models may better reflect local populations but can have more limited generalizability. Importantly, apparent confirmation of a covariate across analyses should be interpreted cautiously when the underlying source populations overlap. For clinical use, model performance in the development dataset alone is insufficient. A model should ideally demonstrate acceptable predictive performance in an external population comparable to the patient being treated and should be linked to an exposure metric with validated clinical relevance. At present, no bedaquiline model fulfills these requirements sufficiently to support routine model-based dose adjustment. Existing models are therefore best used to explain sources of exposure variability, estimate exposure when intensive sampling is impractical, and support hypothesis generation or structured pharmacokinetic interpretation. They should not be used as stand-alone dosing algorithms outside a validated model-informed precision-dosing framework.

## 7 Pharmacokinetics and clinical implications in special populations

### 7.1 Children and adolescents

Direct pediatric pharmacokinetic evidence remains much more limited than current programmatic guidance. WHO supports age- and weight-based bedaquiline dosing across pediatric age groups in appropriate drug-resistant tuberculosis regimens (WHO consolidated guidelines on tuberculosis, 2025). The pivotal TMC207-C211 phase 2 study provided direct exposure-bridging data in 30 children and adolescents aged 5 to <18 years. Age-appropriate dosing produced week-12 weekly bedaquiline exposures within the prespecified adult target range in both age cohorts, supporting pediatric dose selection based on exposure matching rather than simple proportional dose reduction (Moodliar et al., 2021). In 15 children and adolescents aged 6–17 years, an adult population pharmacokinetic model described bedaquiline disposition after allometric scaling and re-estimation of oral bioavailability (Hughes et al., 2022). Estimated bioavailability was 57% of the adult value, and only 3 of 15 participants attained the prespecified adult weekly AUC reference; the small, predominantly adolescent cohort had limited power to identify age- or maturation-dependent effects.

Bedaquiline was generally well tolerated. Ten participants experienced 27 adverse events considered at least possibly treatment-related, but none discontinued bedaquiline. Arthritis and arthralgia were the only severe events, and both resolved without permanent withdrawal. Two participants developed mild Fridericia-corrected QT interval (QTcF) prolongation, with no severe QT prolongation or clinically significant arrhythmia (Hughes et al., 2022).

These data support pediatric dose development through allometric scaling and exposure matching rather than simple proportional dose reduction. They should not be conflated with treatment eligibility: WHO provides pediatric bedaquiline dosing across age groups, whereas the 6-month BPaLM/BPaL regimen and the alternative daily bedaquiline schedule are recommended for adults and adolescents aged ≥14 years (WHO consolidated guidelines on tuberculosis, 2025).

### 7.2 Pregnancy, postpartum and breastfeeding

Evidence is derived principally from a longitudinal study of 13 pregnant women with RR-TB, all of whom were living with HIV and receiving antiretroviral therapy; six completed paired antepartum and postpartum pharmacokinetic sampling (Court et al., 2022). Excluding one participant receiving lopinavir/ritonavir, maternal bedaquiline and M2 concentrations during the elimination phase were approximately 50% lower than predicted by a nonpregnant population model. Concentrations remained below model predictions at a median of 7 weeks postpartum. The study could not determine whether reduced exposure reflected altered clearance, bioavailability or distribution volume and therefore does not support a pregnancy-specific dosing recommendation.

Substantial transfer into breast milk was observed. Effect-compartment modeling estimated equilibrium milk-to-plasma accumulation ratios of 13.6 for bedaquiline and 4.84 for M2, although the delay in milk–plasma equilibration was imprecisely estimated (Court et al., 2022). Estimated infant intake was 0.816 mg/kg/day, approximately two-thirds of the average weight-normalized dose for a 70-kg adult receiving 200 mg three times weekly. Bedaquiline concentrations in one breastfed infant were similar to maternal plasma concentrations, whereas lower concentrations in three nonbreastfed infants were considered likely to reflect residual transplacental exposure.

These findings indicate the potential for substantial infant exposure but do not define its safety or clinical consequences. Likewise, lower maternal total plasma concentrations cannot be equated with clinically important underexposure because unbound concentrations and validated efficacy targets were unavailable.

### 7.3 Hepatic impairment

In an open-label single-dose study, eight participants with moderate hepatic impairment and eight healthy controls received bedaquiline 400 mg. Mean *C*_max_ and AUC_last_ were 14% and 19% lower, respectively, in participants with hepatic impairment (van Heeswijk et al., 2014). Unbound concentrations could not be quantified, and no dedicated pharmacokinetic data are available for severe hepatic impairment. Hepatic-function measures have also not been consistently retained as covariates of apparent clearance in population analyses (Zhu et al., 2021).

Interpretation is complicated by *CYP3A4*-mediated metabolism and extensive protein binding. Changes in total plasma concentration may reflect altered protein binding as well as altered metabolic clearance, and available studies provide no validated exposure threshold for hepatotoxicity. Current evidence therefore supports closer clinical and biochemical monitoring in patients with hepatic dysfunction but does not provide a pharmacokinetic basis for standardized dose adjustment.

### 7.4 Renal impairment

Human data are sparse and inconsistent. In one prospective cohort, higher creatinine clearance was associated with lower bedaquiline AUC_0–24h_, whereas renal-function measures were not retained as significant covariates in other population models (Alghamdi et al., 2021; Zhu et al., 2021). These findings are compatible with the negligible renal elimination of unchanged bedaquiline but provide little information on advanced renal impairment.

The only dedicated disease-state study used a cisplatin-induced renal-impairment model in rats (Gour et al., 2021). Renal impairment reduced AUC_0–24h_ by approximately 30%, whereas ${\text{AUC}}_{0–\infty }$ , apparent clearance, distribution volume and half-life were not significantly altered. The investigators attributed the short-interval difference partly to altered protein binding rather than increased renal elimination. These preclinical findings cannot define human dosing. Clinical data in advanced renal impairment and dialysis remain insufficient, and the effects of uremia on protein binding and nonrenal clearance have not been characterized.

### 7.5 HIV/TB coinfection

HIV infection itself has not emerged as a consistent determinant of bedaquiline pharmacokinetics. HIV status was not retained as a significant covariate in a joint bedaquiline–M2 model (Svensson et al., 2016a). A real-world cohort included only two HIV-positive participants receiving bedaquiline and was not adequately powered for an HIV-specific analysis (Alghamdi et al., 2021). Similarly, concentration–time profiles did not differ qualitatively between three HIV-positive participants and HIV-negative children, but the pediatric study was too small to exclude an HIV- or antiretroviral-specific effect (Hughes et al., 2022).

Earlier population pharmacokinetic analyses have not consistently identified HIV infection as a covariate, but newer direct comparative data suggest that the issue is not fully resolved. In patients with multidrug-resistant tuberculosis, HIV-positive participants had approximately 24% lower bedaquiline AUC_0–22h_ than HIV-negative participants, whereas *C*_max_ was similar (Shenje et al., 2025). The clinical significance of this moderate reduction remains uncertain. More importantly, *CYP3A* induction or inhibition by antiretroviral agents can substantially alter bedaquiline exposure during prolonged treatment. The quantitative effects of efavirenz, nevirapine and lopinavir/ritonavir are considered in Section 8.3. Key evidence on exposure variability, target-site distribution and special populations is summarized in Table 1.

## 8 Drug–drug interactions and clinical management

### 8.1 *CYP3A* inducers

Rifampicin and rifapentine produce the most clearly quantified induction effects. In healthy volunteers, repeated administration of either agent reduced bedaquiline *C*_max_ by approximately 38%–40% and AUC by 53%–59% after a single bedaquiline dose (Winter et al., 2015). Both agents accelerated early M2 formation, nearly doubling M2 *C*_max_, while reducing total M2 exposure through induction of downstream clearance.

Model-based analyses predicted substantially greater effects during chronic coadministration (Svensson et al., 2016b). Rifampicin and rifapentine increased apparent bedaquiline clearance by 4.78-fold and 3.96-fold, respectively, and were predicted to reduce average steady-state exposure by 79% and 75% (Svensson et al., 2015). Simulated dose escalation could restore average parent-drug exposure but produced high early M2 peaks and exaggerated peak–trough fluctuations. Because the safety of these exposures has not been established, empirical bedaquiline dose escalation to overcome rifamycin induction is not recommended.

Rifabutin had a smaller effect on bedaquiline exposure than rifampicin in a randomized healthy-volunteer study (Healan et al., 2018). However, lymphopenia, including severe events, occurred frequently, precluding its assumption as a risk-free substitute. Direct bedaquiline interaction studies with carbamazepine or phenytoin were not identified, and quantitative extrapolation is therefore inappropriate. Potent *CYP3A* inducers should generally be avoided because of the risk of clinically important underexposure.

### 8.2 *CYP3A* inhibitors

*CYP3A* inhibition generally produces smaller short-term effects than rifamycin induction, but its consequences may increase during prolonged coadministration. In a physiologically based pharmacokinetic analysis, ketoconazole increased bedaquiline AUC by approximately 22%, with little change in *C*_max_ (Humphries et al., 2021). Model performance was less reliable for M2, reflecting uncertainty in metabolite disposition.

In a phase 1 study, clarithromycin 500 mg every 12 h for 14 days increased bedaquiline AUC_0–240h_ by approximately 14% after a single 100-mg dose, without materially altering *C*_max_ or *T*_max_ (Kurosawa et al., 2021). However, simulations of 48 weeks of coadministration predicted increases in bedaquiline trough concentrations of 33% at week 24 and 41% at week 48. Thus, modest short-term changes should not be assumed to represent the effect of sustained *CYP3A* inhibition.

In mice, verapamil increased bedaquiline AUC_0–24h_ by 28%–46%, with improved antibacterial activity largely attributable to greater systemic exposure (Xu et al., 2018). Direct quantitative data for itraconazole, fluconazole and erythromycin remain limited. Management should therefore consider inhibitor potency, treatment duration and available alternatives rather than extrapolating from a single short-duration study.

### 8.3 Antiretroviral drugs

Efavirenz illustrates the limitations of single-dose interaction studies for a drug with prolonged disposition. In healthy volunteers, steady-state efavirenz reduced bedaquiline AUC_0–336h_ by 18% without changing *C*_max_ and increased M2 *C*_max_ by 89% (Dooley et al., 2012). Model-based extrapolation to chronic treatment predicted an approximately 52% reduction in average steady-state bedaquiline and M2 exposure (Svensson et al., 2013), indicating that the single-dose study underestimated the likely long-term effect.

In patients with HIV and drug-resistant tuberculosis, nevirapine had little clinically relevant effect, with predicted bedaquiline AUC_0–48h_ increases of 14% at week 10 and 17% at week 24 (Brill et al., 2017). By contrast, lopinavir/ritonavir reduced bedaquiline clearance by approximately 75% and was predicted to increase AUC_0–48h_ by 120% at week 10 and 187% at week 24, with continued parent-drug accumulation (Brill et al., 2017).

These opposing effects preclude a uniform approach to antiretroviral coadministration. Efavirenz may cause clinically important underexposure, whereas ritonavir-boosted regimens may markedly increase and prolong exposure. Proposed model-based dose modifications have not been prospectively validated. Direct evidence for an interaction with dolutegravir is lacking; data concerning dolutegravir and delamanid should not be used to infer the absence of a bedaquiline interaction.

### 8.4 Other antituberculosis drugs and overlapping toxicity

Pharmacokinetic interactions should be distinguished from pharmacodynamic toxicity. Clofazimine was not associated with a statistically significant change in bedaquiline bioavailability or in the shared clearance of bedaquiline and M2, although wide confidence intervals did not exclude a clinically relevant effect (Maartens et al., 2018). In a joint model of delamanid and DM-6705, bedaquiline did not significantly alter their pharmacokinetics (Tanneau et al., 2022). However, the reciprocal effect of delamanid on bedaquiline and M2 was not evaluated.

The principal concern with clofazimine, delamanid and fluoroquinolones is pharmacodynamic additivity of QT prolongation rather than a demonstrated pharmacokinetic interaction (Jin et al., 2023). Evidence involving pretomanid and linezolid has largely addressed regimen-level exposure–response and exposure–toxicity relationships rather than direct bedaquiline interactions and is discussed in Section 9.3.

### 8.5 Clinical management principles

Potent *CYP3A* inducers, particularly rifampicin and rifapentine, should generally be avoided because they markedly reduce bedaquiline exposure and no validated compensation strategy exists. Prolonged strong *CYP3A* inhibition or ritonavir-boosted antiretroviral therapy requires caution because accumulation may be greater than short-duration studies suggest.

When multiple QT-prolonging drugs are combined, electrocardiogram (ECG) and electrolyte monitoring should be intensified, particularly in patients with baseline QT prolongation or other cardiac risk factors. Shared toxicity alone does not establish a pharmacokinetic interaction; exposure changes and pharmacodynamic additivity should be assessed separately.

When an unavoidable interaction creates clinically meaningful uncertainty about exposure, selective pharmacokinetic assessment may help explain the problem, but empirical dose adjustment is not supported. Observed and modeled interactions and their clinical implications are summarized in Table 2, Part A.

**TABLE 2:** Clinically relevant drug-drug interactions and exposure-response evidence for bedaquiline^a,^ ^b^.

Drug(s)	Evidence and mechanism	Effect on exposure	Clinical implication	Key limitation
Part A. Drug-drug interactions				
Rifampicin and rifapentine (Winter et al., 2015; Svensson et al., 2015)	Repeated rifamycin exposure with a single BDQ dose in healthy volunteers; chronic effects simulated. Potent *CYP3A* induction	Observed BDQ *C*_max_ decreased about 38%–40% and partial AUC about 53%–59%. Modeled chronic coadministration reduced average steady-state BDQ/M2 exposure by about 75%–79%	Avoid concomitant use. Empirical BDQ dose escalation to overcome induction is not validated and may produce excessive early M2 exposure	Single-dose BDQ studies; chronic effects and dose-compensation risks were model-predicted
Rifabutin (Healan et al., 2018)	Randomized healthy-volunteer study; steady-state rifabutin with a single 400-mg BDQ dose. Weaker *CYP3A* induction	No statistically significant reduction in overall BDQ exposure ( ${\text{AUC}}_{0-\infty }$ GMR, 0.918; 95% CI, 0.808–1.044)	Rifabutin had a substantially smaller effect on parent-BDQ exposure than rifampicin, but the evidence is insufficient to endorse it as a routine rifamycin substitute. If coadministration is unavoidable, monitor for rifabutin-associated cytopenias	Small healthy-volunteer study with single-dose BDQ; frequent lymphopenia and neutropenia were temporally associated with rifabutin, but the safety findings cannot be generalized to patients with TB.
Efavirenz (Dooley et al., 2012; Svensson et al., 2013)	Healthy-volunteer DDI study plus model-based chronic extrapolation. *CYP3A* induction	Observed BDQ AUC_0-336h_ decreased 18%, but modeled long-term average BDQ/M2 exposure decreased about 50%–52%; M2 *C*_max_ increased 89% in the single-dose study	Prefer a noninducing antiretroviral regimen when feasible. No efavirenz-specific BDQ dose adjustment is prospectively validated	Participants did not have TB; long-term magnitude was model-based
Nevirapine and lopinavir/ritonavir (Brill et al., 2017)	PopPK analysis in people with HIV and DR-TB. Nevirapine has limited induction; ritonavir strongly inhibits *CYP3A*	Nevirapine increased predicted AUC_0-48h_ by only 14%–17%. LPV/r reduced BDQ clearance by about 75% and increased predicted AUC_0-48h_ by 120% at week 10 and 187% at week 24	Nevirapine had little clinically relevant effect. With LPV/r, use alternatives when possible and intensify ECG, electrolyte, and toxicity monitoring	Small observational groups; proposed reduced BDQ regimens with LPV/r are not validated
Ketoconazole and clarithromycin (Humphries et al., 2021; Kurosawa et al., 2021)	Short healthy-volunteer studies; strong or moderate *CYP3A* inhibition, with longer-term effects simulated for clarithromycin	Short-term BDQ AUC increased 22% with ketoconazole and 12%–14% with clarithromycin; simulated clarithromycin coadministration increased *C*_trough_ by 33% at week 24 and 41% at week 48	Do not extrapolate modest single-dose effects to prolonged inhibition. Consider alternatives and enhanced safety monitoring when prolonged coadministration is unavoidable	Short inhibitor exposure or single BDQ doses; long-term accumulation was simulated
Clofazimine, delamanid^c^, and other QT-prolonging drugs (Maartens et al., 2018; Tanneau et al., 2022; Jin et al., 2023)	Secondary PopPK analyses and a prospective QT cohort. The principal concern is overlapping pharmacodynamic QT effects and cumulative regimen-level QT burden rather than a demonstrated PK interaction	Clofazimine did not significantly alter BDQ bioavailability or BDQ/M2 clearance. BDQ did not significantly alter delamanid/DM-6705 PK. QTc increased with the number of QT-prolonging drugs	No PK-based BDQ dose adjustment is supported. Obtain serial ECGs, correct electrolytes, and consider the full regimen-level QT burden	Secondary analyses with wide confidence intervals; the delamanid analysis tested the BDQ-to-delamanid direction only

Evidence source	Exposure metric and setting	Endpoint	Main finding	Interpretation and limitation
Part B. Pharmacodynamic, exposure-response, and exposure-toxicity evidence^d^				
Murine dose-fractionation study (Rouan et al., 2012)	Weekly BDQ/M2 AUC_0-168h_; total weekly dose; *C*_max_ and time above MIC/MBC also evaluated	Lung CFU reduction after 4–6 weeks	Bactericidal activity was similar across once-, twice-, and 5-times-weekly dosing when total weekly dose was matched; AUC best tracked response	Supports AUC as a preclinical PK-PD driver, but provides no human therapeutic target; relative M2 exposure differs between mice and humans
C208 phase IIb PK-PD model (Svensson et al., 2017)	Time-varying weekly average BDQ concentration	Mycobacterial-load decline and week-20 culture conversion	Higher exposure was associated with faster clearance. Model EC50 was 1.42 mg/L; predicted nonconversion declined from 17.1% at half-median to 6.6% at double-median exposure	The EC50 is a model parameter, not a treatment target; older optimized-background regimens and baseline disease factors limit transfer to modern regimens
Shao et al., 2023 (Shao et al., 2023)	Model-estimated AUC_0–24h_/MIC; development cohort n = 55 and prospective validation cohort n = 159	2- and 6-month culture conversion and final treatment success	AUC_0–24h_/MIC thresholds were 175.5 for 2-month conversion, 118.2 for 6-month conversion, and 74.6 for treatment success. Standard dosing achieved PTA >90% for 94% of isolates with MIC ≤0.125 mg/L	Supports adequacy of standard dosing for most susceptible isolates in this cohort, but does not establish MIC-guided dose adjustment. Thresholds were derived and evaluated within the same multicenter study, used 7H11 MICs, omitted M2, and lack independent external validation
Small clinical exposure-response cohorts (Bhatnagar et al., 2024; Mariana et al., 2025)	*C*_max_, *C*_max_/MIC, and AUC_0-24h_ in Indonesian and BEAT-TB India cohorts	Sputum culture conversion	Converters had higher *C*_max_ and *C*_max_/MIC in the Indonesian cohort; BEAT-TB showed accumulation from weeks 8–16 but no defensible threshold	Exploratory only: small PK samples, sparse timing, observational confounding, and MIC variability preclude a validated *C*_max_, AUC, or *C*_max_/MIC target.
Exposure-QT analyses (Tanneau et al., 2021; Vongjarudech et al., 2025; Abdelwahab et al., 2026)	Model-predicted M2, pretomanid, and clofazimine concentrations; alternative QT-correction and joint interaction models	Longitudinal QT/QTcF change	In C208/C209, M2 was the strongest exposure predictor of QTcF prolongation (E_max_, 28.6 ms; EC_50_, 855 ng/mL). Using QTcTBT, an M2 concentration of 326 ng/mL was associated with an estimated 7.01-ms increase. A subsequent competitive-interaction model identified joint concentration-dependent effects of M2, pretomanid, and clofazimine, with a shared maximal effect of 44.5 ms rather than simple additivity	An exposure-related QT effect is supported, but its magnitude depends on the correction method, time-on-treatment effects, and companion drugs. No validated M2 threshold predicts clinically important arrhythmia, and the effects of QT-prolonging drugs should not be assumed to be simply additive
Prospective Georgian QT cohort (Jin et al., 2023)	BDQ AUC, *C*_max_, and *C*_min_ quartiles in 94 participants	Serial QTc during months 1–12	BDQ exposure metrics were associated with QTc during months 1–6, and *C*_max_ remained associated during months 7–12; QTc increased with the number of QT-prolonging drugs	Regimen-level observational evidence; parent BDQ, M2, and companion-drug effects were not separable; no intervention threshold was derived
PRACTECAL-PKPD nested study (Nyang’wa et al., 2026)	Model-derived exposure to BDQ and companion drugs in BPaL, BPaLC, and BPaLM regimens	MGIT killing slope, long-term outcome, and adverse events	Exposure to BDQ or any evaluated companion drug was not associated with between-patient variability in sputum bacterial clearance or long-term outcome. The MGIT-TTP killing slope was approximately 20% steeper with BPaLM than with standard care, whereas BPaL/BPaLC had an approximately 15% less-steep slope. Only linezolid AUC_0–24h_ was associated with grade ≥1 anemia and neutropenia; no other exposure-toxicity relationship was identified	Argues against routine pursuit of single-drug exposure targets within effective multidrug regimens. However, the study was not dose-ranging, included a restricted exposure range, and may have been underpowered to detect correlated drug effects; extreme underexposure cannot be assumed to be harmless

^a^ Observed effects are measured clinical pharmacokinetic findings; modeled effects are simulations or extrapolations.

^b^ No prospectively validated universal BDQ or M2 exposure target or dose-adjustment algorithm is currently available.

^c^ For delamanid, the available analysis primarily evaluated the effect of BDQ on delamanid/DM-6705, not the reverse direction.

^d^ Pharmacodynamic models summarized here were developed for different purposes, including characterization of bacterial killing, exposure–response assessment, QT modeling, regimen simulation, and outcome prediction; their parameter estimates and targets should not be considered interchangeable.

Abbreviations: AUC, area under the concentration-time curve; BDQ, bedaquiline; BPaL, bedaquiline-pretomanid-linezolid; BPaLC, bedaquiline-pretomanid-linezolid-clofazimine; BPaLM, bedaquiline-pretomanid-linezolid-moxifloxacin; CFU, colony-forming unit; CI, confidence interval; CL/F, apparent oral clearance; *C*_max_, maximum concentration; *C*_min_/*C*_trough_, minimum or trough concentration; DDI, drug-drug interaction; DR-TB, drug-resistant tuberculosis; ECG, electrocardiogram; E_max_, maximum modeled effect; EC_50_, concentration producing 50% of the modeled maximal effect; GMR, geometric mean ratio; LPV/r, lopinavir/ritonavir; M2, N-monodesmethyl bedaquiline; MBC, minimum bactericidal concentration; MGIT, mycobacterial growth indicator tube; MIC, minimum inhibitory concentration; PK, pharmacokinetic; PK-PD, pharmacokinetic-pharmacodynamic; PTA, probability of target attainment; QTc, corrected QT interval; QTcF, Fridericia-corrected QT interval; QTcTBT, tuberculosis-specific corrected QT interval; TTP, time to positivity.

## 9 Pharmacokinetic–pharmacodynamic and safety relationships

### 9.1 Pharmacodynamic models and exposure–efficacy relationships

Bedaquiline pharmacokinetic–pharmacodynamic (PK–PD) models address different questions and should not be treated as interchangeable. Preclinical dose-fractionation studies relate systemic exposure to bacterial killing; clinical models link exposure to mycobacterial burden and culture conversion; regimen-level models incorporate interactions among companion drugs; and exposure–toxicity models relate parent drug, M2, and QT-prolonging co-medications to longitudinal QT changes. MIC measures inhibition of growth under standardized conditions, whereas MBC measures bactericidal activity under the assay conditions. Neither directly captures sterilizing activity against slow- or nonreplicating bacilli within heterogeneous tuberculous lesions. Consequently, systemic AUC/MIC or *C*_max_/MIC incorporates isolate susceptibility but does not directly quantify sterilizing exposure at the anatomical site of persistent bacilli.

In a murine dose-fractionation model, bactericidal activity was similar when the same total weekly bedaquiline dose was divided once, twice, or five times weekly, and AUC_0–168h_ most closely tracked lung bacterial killing (Rouan et al., 2012). This supports cumulative exposure as a preclinical PK–PD driver but does not establish a human therapeutic target. AUC_0–168h_ should not be treated as interchangeable with AUC_0–24h_, *C*_min_, or *C*_max_/MIC reported in clinical cohorts.

Clinical pharmacometric analyses have also identified exposure–response relationships. In patients with drug-resistant tuberculosis, higher dynamically updated weekly average bedaquiline concentrations were associated with faster mycobacterial clearance, with a model-estimated half-maximal effective concentration of 1.42 mg/L (Svensson et al., 2017). At week 20, predicted nonconversion was approximately 17%, 12%, and 7% at half, median, and twice the median exposure, respectively. A separate 14-day early bactericidal activity analysis estimated a similar half-maximal concentration of 1.6 mg/L with an onset delay of about 40 h (Lyons, 2022a). These model parameters are not validated treatment targets.

The BEAT-TB India substudy likewise showed accumulation rather than a defensible therapeutic threshold: median AUC_0–24h_ increased from 27.3 μg⋅h/mL at week 8–35.5 μg⋅h/mL at week 16 (Bhatnagar et al., 2024). Intensive pharmacokinetic sampling was available for only 23 and 19 participants at the two visits, respectively.

By contrast, PRACTECAL-PKPD did not identify an independent bedaquiline exposure–response relationship within effective multidrug regimens (Nyang’wa et al., 2026); regimen-level findings are discussed in Section 9.3. No AUC-, concentration-, or MIC-normalized threshold has been sufficiently validated for routine clinical use.

### 9.2 Human exposure–toxicity relationships

Human evidence linking bedaquiline or M2 exposure to toxicity has focused primarily on QT-interval prolongation. In an analysis of 429 participants from the C208 and C209 trials, M2 concentration, rather than parent-bedaquiline concentration, predicted QTcF prolongation (Tanneau et al., 2021). The estimated maximal M2-related increase was 28.6 ms, with a half-maximal effective concentration of 855 ng/mL. Clofazimine and moxifloxacin contributed additional modeled increases of 11.8 and 2.47 ms, respectively.

Reanalysis using a time-varying, tuberculosis-specific correction factor yielded a smaller and more stable estimate (Vongjarudech et al., 2025). At an M2 concentration of 326 ng/mL, representative of the end of loading, predicted QT prolongation was 7.01 ms, indicating that effect estimates depend partly on the heart-rate correction method.

Other studies suggest that QT effects are not attributable exclusively to M2. In a prospective cohort of 94 patients, bedaquiline AUC, C_max_, and *C*_min_ were associated with QTc prolongation, which also increased with the number of QT-prolonging drugs (Jin et al., 2023). However, no clinically significant arrhythmias occurred. A joint concentration–QT model additionally identified concentration-dependent effects of M2, pretomanid, and clofazimine (Abdelwahab et al., 2026).

Collectively, human data support an exposure-related QT effect involving M2, while parent-drug exposure, treatment duration, companion drugs, and QT-correction methods may also contribute. No M2 concentration has been prospectively validated as a clinical toxicity threshold. These human findings should be distinguished from preclinical phospholipidosis. The C208/C209 analysis also found no significant association between bedaquiline or M2 exposure and longitudinal alanine aminotransferase or aspartate aminotransferase concentrations (Tanneau et al., 2021), arguing against a simple concentration-dependent explanation for hepatotoxicity in multidrug regimens.

### 9.3 PK–PD evidence in BPaL- and BPaLM-based regimens

Regimen-level pharmacodynamic models extend single-drug exposure–response analyses by jointly considering regimen composition and pharmacodynamic interactions among companion drugs. This distinction is particularly important for BPaL- and BPaLM-based therapy, in which microbiological response cannot necessarily be attributed to bedaquiline exposure in isolation. A mechanism-based analysis of bedaquiline–pretomanid–pyrazinamide therapy, developed from short-term studies in drug-susceptible pulmonary tuberculosis, showed that regimen-level activity could not be inferred from bedaquiline exposure alone (Lyons, 2022b). Antagonism between bedaquiline and pretomanid was offset by favorable interactions involving pyrazinamide, indicating that overall activity reflected the combined exposures and pharmacodynamic interactions of all regimen components.

A mechanistic BPaL model predicted only minor differences in median time to colony-forming units clearance between bedaquiline 200 mg once daily and the loading–intermittent regimen (Mehta et al., 2024b). It also suggested that lower linezolid doses might preserve most antibacterial activity. However, these findings remain model-based and do not establish equivalent clinical efficacy.

In PRACTECAL-PKPD, BPaLM was associated with an approximately 20% faster modeled sputum bacillary killing rate than standard-of-care therapy, whereas BPaL and bedaquiline–pretomanid–linezolid–clofazimine were associated with approximately 15% slower killing (Nyang’wa et al., 2026). These estimates describe modeled microbiological clearance trajectories and should not be interpreted as direct comparisons of overall clinical efficacy. Exposure to individual drugs did not predict bacterial clearance or treatment outcome. Severe disease was associated with approximately 20% slower bacterial clearance. Among the evaluated agents, only linezolid showed a clinically relevant exposure–toxicity relationship, with higher exposure among patients who developed anemia or neutropenia.

These findings caution against applying single-drug concentration targets directly to BPaL- or BPaLM-based therapy. Regimen composition, pharmacodynamic interactions, disease severity, and companion-drug exposure may be more informative than bedaquiline exposure alone. Relevant evidence and candidate metrics are summarized in Table 2, Part B. The limitations of extrapolating single-drug pharmacokinetics to regimen efficacy are further illustrated by endTB-Q, in which a shortened bedaquiline–delamanid–linezolid–clofazimine strategy did not establish overall noninferiority to the longer control strategy in fluoroquinolone-resistant disease (Guglielmetti et al., 2025).

## 10 Therapeutic drug monitoring and dose optimization

### 10.1 Potential indications for therapeutic drug monitoring

Routine bedaquiline therapeutic drug monitoring (TDM) is not supported because no plasma concentration, AUC, or metabolite threshold has been prospectively validated for dose adjustment. Selective TDM may nevertheless be informative when exposure is uncertain, including in clinically complex pediatric or pregnant patients, suspected malabsorption or nonadherence, major *CYP3A*-mediated interactions, hepatic dysfunction, unexplained treatment failure, prolonged treatment interruption, or unexpected toxicity. These are expert-informed applications rather than validated indications (Alffenaar et al., 2022). Measurements should answer a specific clinical question and be interpreted with dosing history, food intake, adherence, regimen composition, microbiological response, and safety findings. Sampling must match the dosing schedule: during daily dosing, an immediately pre-dose sample is *C*_trough_; during three-times-weekly maintenance, the true trough occurs before the next scheduled dose, often 48 or 72 h after the previous dose. Full-interval AUC_0–τ_ or standardized cumulative exposure may be more coherent than isolated AUC_0–24h_ during intermittent dosing, but no such target is prospectively validated. Figure 2 summarizes a selective assessment framework.

**FIGURE 2:**
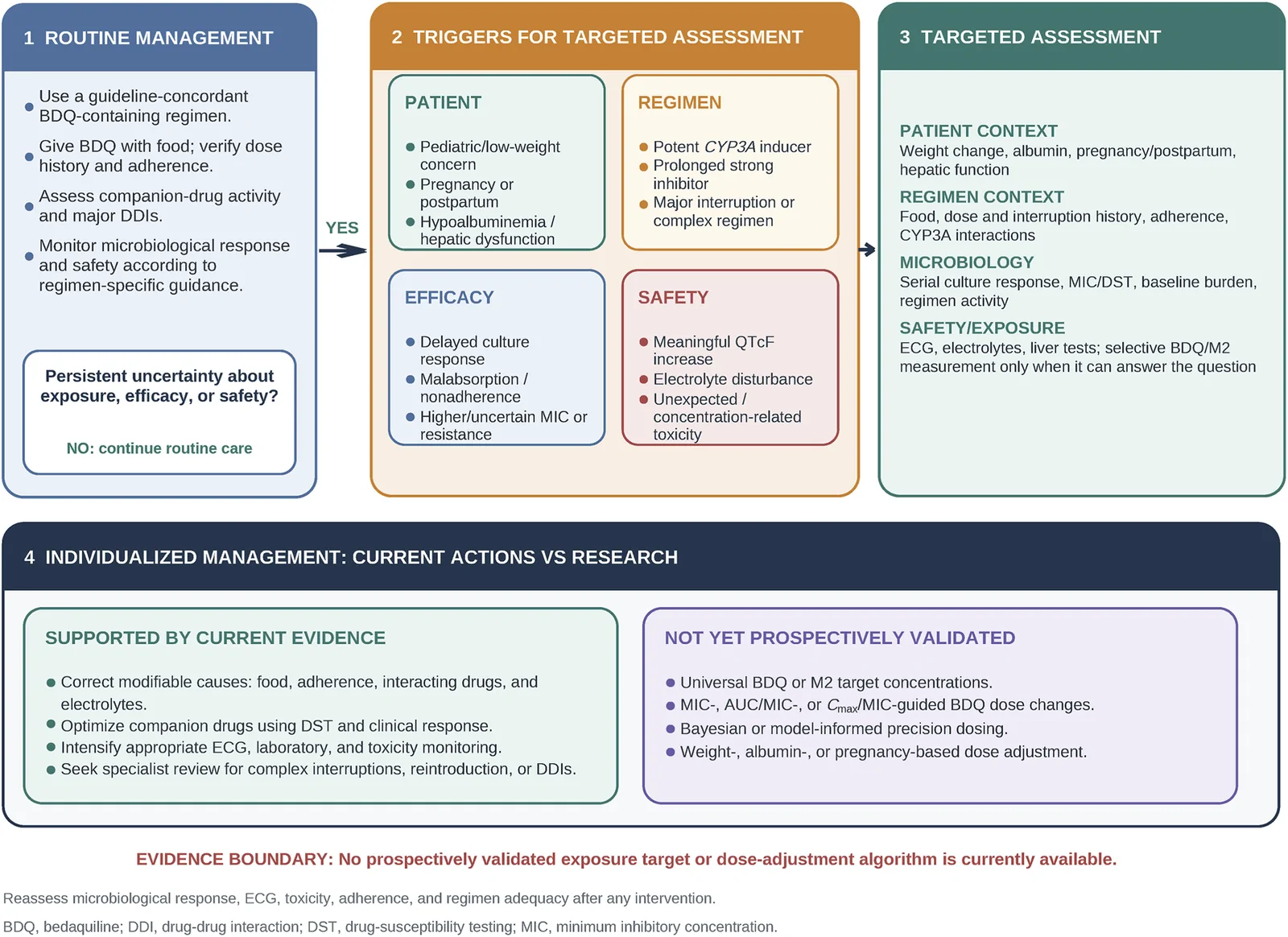
Selective assessment and management framework for individualized bedaquiline therapy.

Analytical feasibility is no longer the principal barrier. A validated liquid chromatography–tandem mass spectrometry assay can quantify bedaquiline and M2 using 10 μL of serum (Alffenaar et al., 2015), and multiplex methods permit simultaneous measurement of other second-line drugs (Kohler et al., 2023). A dried-blood-spot assay has also been validated for bedaquiline, M2, linezolid, levofloxacin, and clofazimine (Kenfack Teponnou et al., 2025). Bedaquiline concentrations in dried blood spots and plasma were strongly correlated (*r* = 0.988), although performance was less robust for M2. These methods facilitate decentralized sampling but do not resolve the absence of validated interpretive targets.

### 10.2 MIC-normalized exposure and its limitations

Susceptibility-normalized indices may be more informative than concentration alone in some settings, but available *C*_max_/MIC and AUC/MIC evidence remains exploratory and has not yielded a prospectively validated bedaquiline target (Rouan et al., 2012; Shao et al., 2023; Mariana et al., 2025).

In a Chinese population pharmacokinetic study, data-driven AUC_0–24h_/MIC cutoffs of 175.5 for 2-month culture conversion, 118.2 for 6-month conversion, and 74.6 for treatment success were identified in a development cohort of 55 patients and evaluated in a prospective cohort of 159 patients (Shao et al., 2023). These cutoffs remain study-specific: they were derived within a particular population, regimen, MIC method, and modeling framework, and they should not be extrapolated to AUC_0–τ_/MIC, weekly AUC/MIC, or lesion sterilization without independent prospective validation. This limitation is particularly relevant for bedaquiline because phenotypic susceptibility results and interpretive thresholds are method-dependent, requiring standardized and validated testing procedures (Kaniga et al., 2020).

MIC-informed dose adjustment therefore remains investigational. Dose escalation should not be based solely on a low AUC/MIC or *C*_max_/MIC ratio because higher parent-drug exposure may also increase M2 accumulation and QT risk.

### 10.3 Model-informed individualized dosing

Population pharmacokinetic models permit exposure estimation when intensive sampling is impractical. Existing models incorporate body size, albumin, treatment-related physiological changes, and *CYP3A*-mediated interactions, although covariate effects differ among populations (McLeay et al., 2014; Svensson et al., 2016a; Zhu et al., 2021; Zou et al., 2022; Shao et al., 2023).

Mechanistic PK–PD models further link exposure to bacterial clearance and toxicity. An interactive platform integrating bedaquiline and M2 pharmacokinetics with Mycobacteria Growth Indicator Tube time-to-positivity, QTcF, and long-term outcomes compared alternative dosing and reloading strategies (Lin et al., 2026). The ZeNix once-daily regimen was predicted to produce approximately 50% lower median bedaquiline and M2 average concentrations at week 2 than regimens using the registered or standard loading phase. This reduction was associated with 11% fewer simulated patients having negative cultures at week 2 and 5.8% fewer achieving culture conversion by week 8, whereas predicted QTcF distributions were similar.

A multistate model based on 402 participants from the C208 and C209 trials linked faster early bacterial clearance and lower baseline burden to culture conversion, whereas greater modeled burden at treatment completion was associated with recurrence through 120 weeks (Lin et al., 2024). However, its incremental predictive value was limited, and it was developed for prognosis and trial simulation rather than individual dose adjustment.

No Bayesian dosing procedure or limited-sampling strategy has been prospectively validated for routine bedaquiline dose selection. Current models are better suited to exposure estimation, interpretation, and hypothesis testing than to independent dose adjustment.

## 11 Discussion

### 11.1 What current pharmacokinetic evidence can inform clinical decisions

Several pharmacokinetic principles are sufficiently established to guide current management without requiring a concentration target. Bedaquiline should be administered with food; potent *CYP3A* inducers, especially rifampicin and rifapentine, should generally be avoided; prolonged strong *CYP3A* inhibition requires caution; and combinations with other QT-prolonging drugs warrant intensified ECG and electrolyte monitoring. These measures address reproducible determinants of exposure or safety rather than attempting to titrate a patient to an unvalidated concentration.

Pharmacokinetic assessment may still be useful when a specific source of uncertainty exists, for example, suspected malabsorption or nonadherence, major drug–drug interactions, prolonged interruption, unexpected toxicity, treatment failure, or a poorly characterized special population. Its immediate clinical value is to explain unusual systemic exposure, not to prove adequate target-site exposure or sterilizing activity. Plasma TDM cannot establish that effective concentrations have been achieved throughout heterogeneous lesions, particularly poorly vascularized caseum.

### 11.2 Where the evidence remains conflicting

The major uncertainty is not whether bedaquiline exposure varies between patients, but whether the sources and consequences of that variability are sufficiently reproducible to guide intervention. Population pharmacokinetic models consistently demonstrate substantial interindividual variability, yet covariate relationships have differed across cohorts. Body weight and albumin have the strongest repeated physiological support, but neither has shown sufficiently uniform effects to justify a general dose-adjustment rule. Associations involving age, sex, renal function, race, biochemical markers, and pharmacogenetic variants are less consistent or remain population-specific. Some apparent replication also arises from analyses of overlapping clinical-trial datasets and therefore cannot be regarded as independent confirmation.

Exposure–response evidence is similarly heterogeneous. Preclinical dose-fractionation studies support cumulative exposure as an important determinant of bacterial killing, and several clinical pharmacometric analyses have identified associations between bedaquiline exposure and microbiological response. However, smaller observational studies have reported different candidate metrics and thresholds, whereas PRACTECAL-PKPD did not identify an independent bedaquiline exposure–response relationship within effective multidrug regimens. Exposure–toxicity analyses more consistently support a concentration-related QT effect, particularly involving M2, but estimates vary according to QT-correction method, treatment duration, parent-drug exposure, and concomitant QT-prolonging agents. These discrepancies argue against reducing bedaquiline pharmacology to a single concentration, covariate, or exposure threshold.

### 11.3 What cannot yet be inferred from available pharmacokinetic data

Several clinically important inferences remain unsupported. First, a measured plasma concentration cannot presently be translated directly into an individualized dose recommendation because no universally accepted bedaquiline, M2, AUC, or susceptibility-normalized exposure target has been prospectively validated. Second, statistical identification of a covariate in a population model does not establish that modifying dose according to that covariate improves efficacy or safety. Third, apparent adequacy of systemic exposure should not be equated with adequate sterilizing exposure at the site of disease. MIC remains essential for susceptibility and resistance assessment, but MIC-normalized plasma indices combine only two components of drug action, systemic exposure and *in vitro* susceptibility. They do not directly incorporate lesion penetration, local binding, bacterial metabolic state, or the markedly different microenvironments encountered within cellular and caseous compartments. MBC and other bactericidal measures address bacterial killing more directly than MIC, but even these *in vitro* measures cannot fully reproduce the conditions encountered by slowly replicating or nonreplicating bacilli within avascular caseum.

This limitation defines the boundary of plasma TDM. Bedaquiline can accumulate in plasma, cells, and vascularized lesion regions while penetrating deep caseum much more slowly (Bustion et al., 2025; Mehta et al., 2023; Karakitsios et al., 2025). Accordingly, an apparently adequate plasma AUC or AUC/MIC should not be interpreted as proof of lesion sterilization.

Slow lesion penetration and prolonged persistence may also create periods of suboptimal target-site exposure during treatment or the post-treatment tail. Translational data raise the possibility that such windows could favor selection of resistant subpopulations (Bustion et al., 2025). This remains a mechanistically plausible, translationally supported concern rather than a prospectively quantified clinical risk.

### 11.4 Requirements for precision dosing

A transition from pharmacokinetic characterization to precision dosing will require several linked developments. Sampling first needs to be standardized with respect to dose timing, food intake, treatment phase, and dosing schedule, because measurements obtained during loading, daily maintenance, and intermittent maintenance are not directly interchangeable. Practical limited-sampling strategies and robust assays for both bedaquiline and M2 will be necessary if exposure estimation is to move beyond specialist pharmacology centers.

Second, the biological meaning of measured exposure must be strengthened. Standardized susceptibility testing, measurement of unbound bedaquiline and M2, and better characterization of target-site exposure are required to establish whether systemic exposure reflects concentrations relevant to bacterial killing and toxicity. Special populations, including younger children, pregnant and postpartum patients, and patients with severe hepatic or renal dysfunction, require substantially stronger pharmacokinetic evidence.

Third, pharmacometric models intended for clinical dosing require independent external validation in populations comparable to those in which they will be applied. Future models should integrate bedaquiline and M2 exposure with susceptibility, companion-drug exposure, disease characteristics, and longitudinal safety and microbiological outcomes rather than treating bedaquiline concentration in isolation. Finally, prospective intervention studies must demonstrate that TDM- or model-guided dose modification improves clinically meaningful outcomes beyond careful management of adherence, drug interactions, and toxicity. Until these requirements are met, precision dosing should remain an investigational strategy rather than a justification for empirical departure from validated fixed-dose regimens.

## 12 Conclusion

Bedaquiline has complex and highly variable pharmacokinetics, but several principles already inform clinical use: administration with food, avoidance of potent *CYP3A* induction, caution with prolonged inhibition, and monitoring of cumulative QT risk. Selective pharmacokinetic assessment may help when a defined source of exposure uncertainty exists, but no plasma, M2, AUC, or susceptibility-normalized target has been prospectively validated for routine dose adjustment. Population and PK–PD models are most useful for explaining variability and testing dosing hypotheses. Precision dosing will require standardized exposure assessment, validated pharmacodynamic targets, externally validated models, and prospective evidence that exposure-guided intervention improves clinically meaningful outcomes.
